# Yeast species in the respiratory samples of COVID-19 patients; molecular tracking of *Candida auris*

**DOI:** 10.3389/fcimb.2024.1295841

**Published:** 2024-04-19

**Authors:** Faezeh Rouhi, Sajedeh Soltani, Somayeh Sadeghi, Elahe Nasri, Mahnaz Hosseini, Safiyeh Ghafel, Shima Aboutalebian, Hamed Fakhim, Hossein Mirhendi

**Affiliations:** ^1^ Department of Medical Parasitology and Mycology, School of Medicine, Isfahan University of Medical Sciences, Isfahan, Iran; ^2^ Infectious Diseases and Tropical Medicine Research Center, Isfahan University of Medical Sciences, Isfahan, Iran; ^3^ Mycology Reference Laboratory, Research Core Facilities Laboratory, Isfahan University of Medical Sciences, Isfahan, Iran

**Keywords:** yeast species, respiratory mycobiome, COVID-19, *Candida auris*, colonization

## Abstract

**Introduction:**

Although the existence of *Candida* species in the respiratory tract is often considered commensal, it is crucial to recognize the significance of *Candida* colonization in immunocompromised or COVID-19 patients. The emergence of *Candida auris* as an emerging pathogen further emphasizes the importance of monitoring yeast infection/colonization, particularly in COVID-19 patients.

**Methods:**

In this study, respiratory samples mainly from COVID-19 patients, primarily those suspected of having a fungal infection, were cultured on Sabouraud dextrose agar plates and the yeast colonies were identified using a two-step multiplex PCR method. The samples suspected of *C. auris* underwent specific nested PCR followed by sequence analysis.

**Results:**

A total of 199 respiratory samples were collected from 73 women and 126 men, ranging in age from 1.6 to 88 years. Among the patients, 141 had COVID-19, 32 had cancer, 5 were hospitalized in ICU, 2 had chronic obstructive pulmonary disease)COPD(, and others were patients with combination diseases. From these samples, a total of 334 yeast strains were identified. *C. albicans* (n=132, 39.52%) was the most common species, followed by *C. tropicalis* (n=67, 20%), *C. glabrata* (n=56, 16.76%), *C. krusei* (n=18, 5.4%), *C. parapsilosis* (n=17, 5.08%), *Saccharomyces cerevisiae* (n=10, 3%), *C. kefyr* (n=9, 2.6%), *C. dubliniensis* (n=7, 2.1%), *C. lusitaniae* (n=5, 1.5%), *C. auris* (n=3, 0.9%), *C. guilliermondii* (n=2, 0.6%), *C. rugosa* (n=1, 0.3%), *C. intermedia* (n=1, 0.3%), and *Trichosporon* spp. (n=1, 0.3%). *C. auris* was detected in a patient in ICU and two COVID-19 patients. While its presence was confirmed through sequence analysis, our extensive efforts to isolate *C. auris* were unsuccessful.

**Conclusion:**

While *C. albicans* colonization remains prevalent, our study found no evidence of *Candida* lung infection. Since the role of *Candida* colonization in airway secretions remains ambiguous due to limited research, further studies are imperative to shed light on this matter.

## Introduction

Fungal infection and colonization emerged as significant clinical challenges in recent decades (Delisle et al., 2008). Among nosocomial infections, fungi account for 8-10% of cases, with *Candida* species responsible for 80% of them (Pendleton et al., 2017), particularly in the intensive care unit (ICU) (El-Ebiary et al., 1997). As of October 25, 2022, the World Health Organization (WHO) issued a list of 19 fungal priority pathogens identified as posing the greatest threat to public health, with six *Candida* species (*C. albicans, C. parapsilosis, C. glabrata, C. auris, C. tropicalis*, and *C. krusei*) included in this prioritization (Parums, 2022). *Candida* species are commonly found in the normal microbial flora of the human body and have historically been considered part of the oral microbiota in healthy individuals. They can also be present in the sputum of 20-55% of healthy subjects (Terraneo et al., 2016). Studies have indicated that the presence of *Candida* species in respiratory specimens of immunocompetent patients without concurrent infection is clinically insignificant, as it typically represents colonization rather than infection (Azoulay et al., 2006; Terraneo et al., 2016). However, in immunocompromised patients, *Candida* isolation from sputum, endotracheal aspirates, bronchoscopic samples, percutaneous lung needle aspirates, and lung tissue may rarely indicate invasive pneumonia instead of colonization (El-Ebiary et al., 1997). Factors such as mucus plugging in the lungs, prolonged antibiotic treatment, corticosteroid use, surgically implanted catheters, and longer hospital stays for immunocompromised patients predispose to *Candida* colonization (Baradkar et al., 2009; Muthig et al., 2010).

Although bronchial *Candida* colonization does not directly lead to increased mortality, it is associated with longer durations of ICU and hospital stays, elevated management costs, and poorer outcomes (Azoulay et al., 2006). Furthermore, *Candida* colonization in the lower respiratory tract (RT) or hypersensitivity reactions in different areas have been identified as independent risk factors for the presence of multidrug-resistant bacteria and the development of ventilator-associated pneumonia caused by *Pseudomonas aeruginosa* (Terraneo et al., 2016). Therefore, it is crucial to consider the potential significance of these commensal fungi when they are repeatedly and clearly detected at infection sites. Despite controversies surrounding the diagnosis of pulmonary candidiasis, the definitive diagnosis still relies on the histological presentation of yeast in lung tissue accompanied by inflammation (El-Ebiary et al., 1997), and the value of culturing respiratory specimens for diagnosing *Candida* pneumonia remains unclear. The Clinical Practice Guideline for the Management of Candidiasis does not recommend treatment when *Candida* species are isolated from RT secretions (Pappas et al., 2016). However, it is proposed that the presence of *Candida* colonization in the bronchial area, along with colonization of multiple body sites, should be considered as a risk factor for systemic candidiasis (Azoulay et al., 2006).

*Candida auris*, an emerging pathogen categorized in the WHO critical priority group (Parums, 2022), has been associated with varying levels of antifungal resistance and rapid spread in intensive care settings, leading to widespread nosocomial outbreaks worldwide. Its global presence and recent cases reported in Iran have been demonstrated (Abastabar et al., 2019; Mirhendi et al., 2022; Safari et al., 2022). Accurately identifying this organism poses challenges, which have hindered our understanding of its scope and created uncertainty about its prevalence, transmission, and environmental niches. This highlights the urgent need for ongoing research to address its impact on mortality and healthcare systems. During the recent viral pandemic, the isolation of *C. auris* in COVID-19 patients with acute respiratory distress syndrome (ARDS) has raised worldwide concern. This association has been linked to stays in the ICU, mechanical ventilation, bacterial infections, and the use of immunomodulators such as corticosteroids as risk factors for colonization or infection. It is crucial to identify *C. auris*, even as a colonizer, as candidemia can arise from colonization. Reports of colonization or co-infection by *C. auris* have been documented in COVID-19 patients in several countries, including Italy, Lebanon, the USA, China, Brazil, Colombia, and Spain (Pan American Health Organization/World Health Organization, 2016; Chowdhary et al., 2020; Allaw et al., 2021; Corcione et al., 2022).

There is a dearth of studies specifically examining the importance and role of *Candida* colonization in airway secretions, thus leaving this matter unresolved. Consequently, additional research is required to elucidate the role of *Candida* species, which are generally considered to have a low pathogenicity. The objectives of this study were to assess the frequency of pulmonary yeast colonization in respiratory samples, to identify the yeast isolated from pulmonary specimens, particularly from COVID-19 patients, and to gain insights into their colonization profile. *C. auris* colonization in the patients was also emphasized owing to its clinical significance.

## Materials and methods

### Samples

Between March 2020 and March 2023, respiratory samples including tracheal aspiration (TA), sputum, and bronchoalveolar lavage (BAL) fluid, were collected from patients hospitalized in referral hospitals in Isfahan, Iran. The majority of the samples were from COVID-19 patients who exhibited pulmonary symptoms suggestive of fungal infections, and a smaller number of samples were obtained from immunocompromised patients without COVID-19 infection. Among the patients, there were cases of COVID-19 (n=141), cancer (n=32), ICU admission (n=5), COPD (n=2), and the others were patients with combination diseases (**Table 1**). The study protocol was approved by the ethics committee of the Isfahan University of Medical Science (IR.MUI.MED.REC.1400.448).

**Table 1:** Distribution of the yeast species in respiratory specimens (BAL, sputum, and TA) among diverse patient groups, including patients with COVID-19, cancer, ICU-hospitalized, and COPD.

Yeast species	COVID-19 only (n=141)	Cancer (n=32)	ICU (n=5)	COPD (n=2)	COVID-19 and ICU (n=13)	COVID-19 and cancer (n=4)	COVID-19 and COPD (n=1)	ICU and cancer (n=1)	Total (TA/Sputum/BAL)
*C. albicans*	88	27	4	1	8	3	–	1	132 (65/50/17)
*C. tropicalis*	45	10	2	1	6	2	1	–	67 (37/23/7)
*C. glabrata*	33	15	4	–	1	2	–	1	56 (28/23/5)
*C. krusei*	10	3	–	–	3	2	–	–	18 (13/5/-)
*C. parapsilosis*	14	1	–	–	2	–	–	–	17 (10/4/3)
*C. kefyr*	7	2	–	–	–	–	–	–	9 (3/4/2)
*C. dubliniensis*	6	1	–	–	–	–	–	–	7 (4/2/1)
*C. lusitaniae*	3	1	–	–	1	–	–	–	5 (2/2/1)
*C. auris*	3	–	–	–	–	–	–	–	3 (2/1/-)
*C. guilliermondii*	2	–	–	–	–	–	–	–	2 (-/2/-)
*C. rugosa*	1	–	–	–	–	–	–	–	1 (1/-/-)
*C. intermedia*	1	–	–	–	–	–	–	–	1 (-/1/-)
*Saccharomyces cerevisiae*	8	–	–	–	2	–	–	–	10 (3/5/2)
*Trichosporon* spp	1	–	–	–	–	–	–	–	1 (1/-/-)
*C. famata*	2	–	–	–	–	–	–	–	2 (2/-/-)
*M. capitatus*	2	–	–	–	–	–	–	–	2 (-/2/-)
*Nakaseomyces glabratus*	1	–	–	–	–	–	–	–	1 (1/-/-)
Total	227	60	10	2	23	9	1	2	334 (172/124/38)

### Mycological methods

As part of the routine diagnostic procedure, all samples were plated onto two Sabouraud dextrose agar plates supplemented with 0.5% chloramphenicol. The plates were then incubated at both 25°C and 37°C and monitored daily for significant fungal growth. For this study, yeast colonies that developed on the plates were harvested and subcultured into a fresh medium. The resulting pure colonies were suspended in a 30% glycerol solution and stored in a -20°C freezer until further analysis.

### Molecular identification of the isolates

The DNA extraction from the yeast isolates was carried out using the boiling method (Silva et al., 2012; Salehipour et al., 2021). In summary, colonies were submerged in 50 µl of sterile distilled water and boiled for 20 minutes in a water bath. After centrifugation at 5000 rpm for 10 minutes, the supernatants were stored at -20°C until being used as DNA templates for PCR.

For species identification of the yeast isolates, a stepwise multiplex PCR known as YEAST PLEX was performed as previously described (Aboutalebian et al., 2022). Accordingly, Tube A was utilized for identifying *C. albicans*, *C. dubliniensis*, *C. parapsilosis*, *C. glabrata* (*Nakaseomyces glabratus*), *C*. *tropicalis*, *C. krusei* (*Pichia kudriavzevii*), *C. kefyr* (*Kluyveromyces marxianus*), and *C. auris*; Tube B was used for identifying *C. guilliermondii* (*Meyerozyma guilliermondii*), *C. rugose* (*Diutina rugosa*), *C. intermedia*, *C. lusitaniae* (*Clavispora lusitaniae*), and *C. norvegensis* (*Pichia norvegensis*)*;* and Tube C was employed for identifying *Cryptococcus neoformans*, *Rhodotorula mucilaginosa*, *Trichosporon* spp., and *Saccharomyces cerevisiae*. To identify the rare yeasts that were not covered by YEAST PLEX, a pan-fungal PCR reaction was conducted amplifying the entire internal transcribed spacer (ITS1-5.8SrDNA-ITS2) by using well-known universal primers ITS1 and ITS4, as described previously (White et al., 1990), and the resulting PCR products were purified and sequenced (Core Facilities Research Laboratory, Isfahan, Iran) followed by analysis using the BLAST platform (https://blast.ncbi.nlm.nih.gov/Blast.cgi) for species identification.

### Specific detection of *Candida auris*

**Figure 1:**
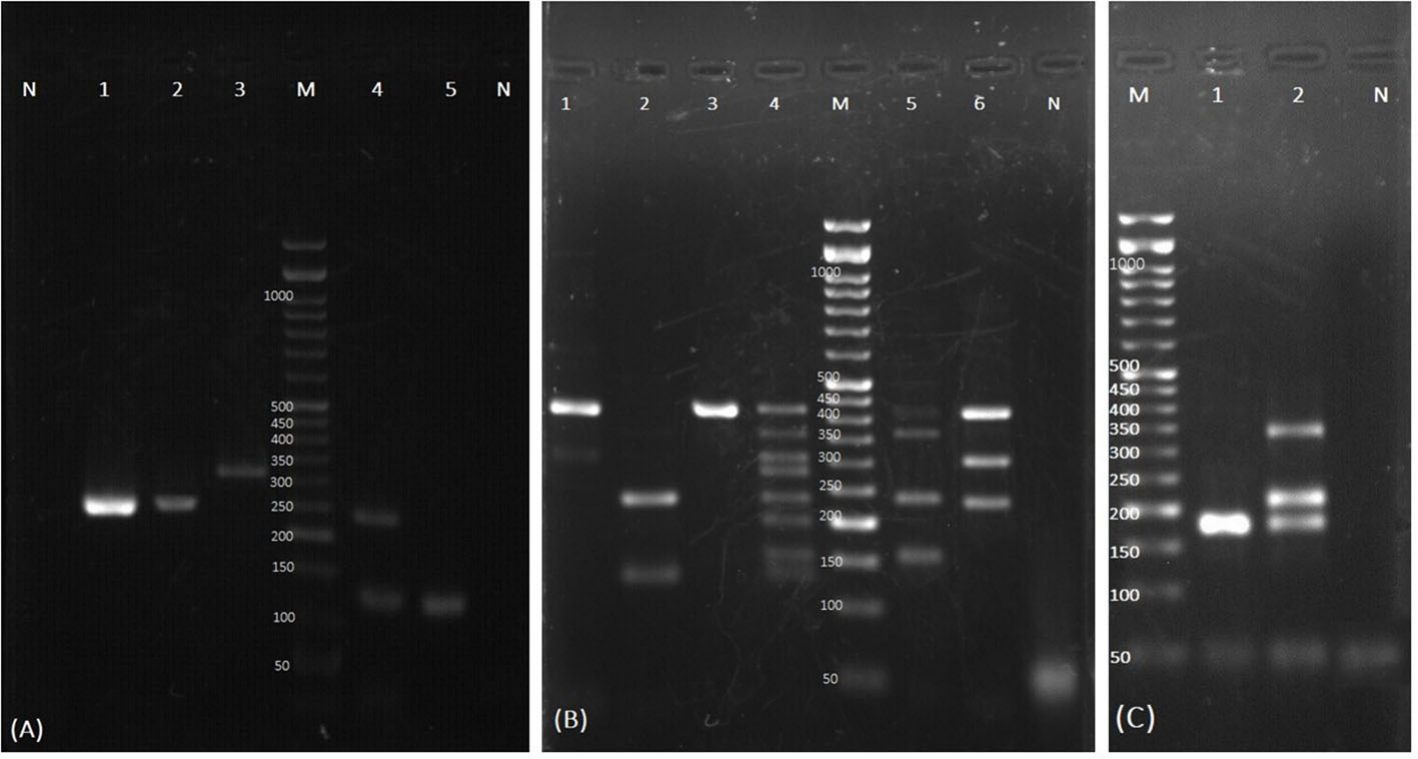
Examples of agarose gel electrophoresis of PCR products for identification of the yeast colonies isolated from respiratory samples. **(A)** Test tube specific for detection of *C. auris*. Lane N: negative control of nested PCR; Lane 1 and 2: *C. auris* with specific final product (220 bp); Lane 3: first round of nested PCR product of *C. auris* (312 bp); Lane M: 50-bp size marker. The yeasts identified by Tube C. are shown in Lane 4,5. Lane 4: *Trichosporon* spp., *Saccharomyces cerevisiae*; Lane 5: *Saccharomyces cerevisiae*; Lane N: negative control of Tube C. **(B)** The yeasts identified by Tube A: Lane 1: *C. albicans* and *C. parapsilosis*; Lane 2: *C. glabrata* and *C. tropicalis*; Lane 3: *C*. *albicans*; Lane 4: Home made size marker for yeast identification; Lane M: commercial 50-bp size marker; Lane 5: *C. albicans*, *C. dubliniensis*, *C. glabrata*, and *C. krusei*; Lane 6: *C. albicans*, *C. parapsilosis*, and *C*. *glabrata*; Lane N: negative control. **(C)** The yeasts identified by Tube B. Lane M: 50-bp size marker; Lane 1: *C. lusitaniae*; Lane 2: *C. guilliermondii*, *C. intermedia*, and *C. lusitaniae*; Lane N: negative control.

To confirm the presence of *C. auris* observed as the faint specific bands in the YEAST PLEX test of certain yeast isolates, a PCR test was performed using universal ITS1 and ITS4 primers, revealing bands of approximately 400 bp, suggesting the possibility of *C. auris* presence. Furthermore, a nested PCR targeting a part of the ITS region was employed. In the first stage of amplification, the external primers 5’-ATTTTGCATACACACTGATTTGG-3’ and 5’-CGACAACAAAACGAAAAAAAGCG-3’ were used to amplify a 312 bp fragment. In the second stage, the internal primers 5’-AACTAACCCAACGTTAAGTTCAAC-3’ and 5’-AACGCCACCGCGAAGATT-3’ were used, yielding a final fragment of 220 bp (Aboutalebian et al., 2021; Aboutalebian et al., 2022) (**Figure 1A**). The first round of PCR included 7.5 µl of 2x premix (Master Mix Red, Ampliqon, Denmark), 0.5 µM primers, and 2 µl DNA template in a total volume of 15 µl, and the thermal conditions were 95°C for 5 min, followed by 35 cycles of 95°C 15 s, 60°C 30 s, and 72°C 30 s. The second PCR consisted of 7.5 µl of 2x premix, 0.33 µM primers, and 2 µl of 1/50 diluted first PCR product, to a final volume of 15 µl, and the reaction conditions included 95°C 5 min, followed by 30 cycles of 95°C 15 s, 60°C 30 s, and 72°C 20 s. Five microliters of the PCR product were electrophoresed, stained, and visualized under ultraviolet light. For further confirmation, the PCR products from positive samples underwent sequencing and BLAST analysis.

### Attempts to isolate *C. auris*

In an attempt to isolate *C. auris* colonies from those colonies that were positive for *C. auris* in molecular tests, selective mediums were used: 1) Sabouraud broths (Merck. Germany) with 10% sodium chloride and 2% mannitol was incubated at 42°C and shacked at 80 rpm for 24 h; 2) Sabouraud broths with 2% of Dulcitol and 4, 8, and 16 μg/mL fluconazole (Rossi et al., 2023) was incubated at 42°C for up to 10 days without agitation; 3) and yeast extract peptone dextrose agar with 12.5% sodium chloride and 9 ml of 9 mM/liter Iron (II) sulfate solution was mixed and incubated for one week at 35°C (Das et al., 2021).

## Results

A total of 199 respiratory samples, including 95 TA, 76 sputum, and 28 BAL were included in this study. The patients consisted of 73 women and 126 men, with ages ranging from 1.6 to 88 years and an average age of 56.

A total of 334 yeast strains were identified from the cultures, predominantly from TA samples, and subjected to PCR-based identification. The most frequently identified species were: *C. albicans* (n=132, 39.52%), *C. tropicalis* (n=67, 20%), and *C. glabrata* (n=56, 16.76%), followed by *C. krusei* (n=18, 5.4%), *C. parapsilosis* (n=17, 5.08%), *Saccharomyces cerevisiae* (n=10, 3%), *C. kefyr* (n=9, 2.6%), *C. dubliniensis* (n=7, 2.1%), *C. lusitaniae* (n=5, 1.5%), *C. auris* (n=3, 0.9%), *C. guilliermondii* (n=2, 0.6%), *C. rugosa* (n=1, 0.3%), *C. intermedia* (n=1, 0.3%), and *Trichosporon* spp. (n=1, 0.3%) (**Figures 1A–C**). Five yeast isolates tested negative with the YEAST PLEX assay, and therefore they were subjected to DNA amplification using ITS universal primers followed by sequencing. They were identified as *C. famata* (*Debaryomyces hansenii*) (n=2, 0.6%), *Magnusiomyces capitatus* (*Geotrichum capitatum*) (n=2, 0.6%) (accession numbers in GenBank is OR600243), and *Nakaseomyces glabratus* (n=1, 0.3%). Although the multiplex PCR system used in this study includes specific primers for the identification of *C. norvegensis*, *Cryptococcus neoformans*, and *Rhodotorula mucilaginosa*, none of these isolates were detected. The distribution of *Candida* species in different clinical samples and the underlying conditions of the patients are presented in **Table 1**.

Within our samples, 109 patients were colonized by one species of *Candida*, 56 patients by two species, and 27 patients by three species. Interestingly, in seven cases four different *Candida* species were identified. Additionally, as shown in **Table 2**, We observed nine cases (4.5%) of co-existence of common *Candida* species (*C. albicans*, *C. glabrata*, *C. tropicalis*, *C. krusei*, *C. dubliniensis*) with other fungi. Among these cases, *A. niger* (n=2), *A. flavus* (n=2), and *A. fumigatus* (n=1) were isolated in 5 samples, confirmed through direct microscopy, culture, and PCR identification. Also, Among the patients with *Candida* species colonization, 5 cases had co-existence with *Pneumocystis*, as documented by specific nested PCR (Matouri et al., 2023). Out of 9 patients with co-occurrence infections with other fungi, one of the patients had a three-fungi isolation of *A. fumigatus*, *Pneumocystis*, and *C. albicans*.

**Table 2:** Concurrent pulmonary fungal infection along with yeast colonization in some respiratory samples examined in this study.

	Gender	Age (year)	Underlying disease	Clinical samples	Yeast species	Isolated mold species	PCR-detected Pneumocystis jirovecii
1	F	69	COVID-19	TA	*C. albicans/C. glabrata/C. tropicalis*	*A. flavus*	Negative
2	F	44	COVID-19	Sputum	*C. dubliniensis*	*A. niger*	Negative
3	M	61	COVID-19	Sputum	*C. albicans/C. glabrata/C. krusei*	*A. flavus*	Negative
4	M	36	COVID-19	Sputum	*C. albicans*	*A. niger*	Negative
5	M	56	COVID-19	Sputum	*C. albicans*	*A. fumigatus*	Positive
6	F	1.5	COVID-19	BAL	*C. albicans*	Negative	Positive
7	M	67	COVID-19/ICU	TA	*C. albicans/C. glabrata/C. tropicalis*	Negative	Positive
8	F	76	COVID-19	TA	*C. albicans/C. glabrata/C. krusei*	Negative	Positive
9	M	78	COVID-19	TA	*C. albicans*	Negative	Positive

M, male; F, female; TA, tracheal aspirate; BAL, bronchoalveolar lavage fluid.

By utilizing *C. auris*-specific nested PCR, we were able to detect *C. auris* in one ICU-hospitalized patient and two COVID-19 patients, as outlined in **Table 3**. Sequence analysis of PCR products confirmed the presence of *C. auris* (accession numbers in GenBank are: OR600362, OR600363, OR600282), however, in spite of multiple mycological attempts made to isolate the colony, none of the selective cultures yielded positive results for *C. auris*.

**Table 3:** Yeast species profiles of three patients in whom *Candida auris* was molecularly detected as a concurrent species.

	Gender	Age (year)	Underlying disease	Clinical sample	identified yeast species			
1	F	87	COVID-19	TA	*C. albicans*	*C. dubliniensis*	*C. glabrata*	
2	M	76	COVID-19	TA	*C. albicans*	*C. glabrata*	*C. krusei*	*C. tropicalis*
3	F	54	ICU	Sputum	*C. albicans*	*C. glabrata*		

F, female; M, male; TA, tracheal aspiration; BAL, bronchoalveolar lavage fluid; ICU, intensive care unit.

## Discussion

As a member of the mycobiome in the human body, *Candida* species can colonize various sites, including the mucosal surfaces of the mouth and vagina, RT, skin folds, and gastrointestinal tract in both healthy and immunocompromised individuals (Caramalac et al., 2007). Microbial colonization plays a significant role in the development of secondary infections that can complicate pulmonary disorders. In immunocompromised patients, asymptomatic colonization of *Candida* species in the RT may be associated with poor outcomes and could potentially alter the antibiotic resistance patterns of pathogenic bacteria through the formation of polymicrobial biofilms. However, the significance of *Candida* colonization in the RT remains a subject of controversy (Liu et al., 2021; Froidefond et al., 2023). In this study, we conducted an analysis of the RT yeast mycobiome by culturing respiratory samples from patients with COVID-19, COPD, and cancer. The results revealed that *Candida* species were the dominant members in the heterogeneous patient populations, with *C. albicans* being the most frequently identified species (39.52%), highlighting the common occurrence of *C. albicans* in hospitalized patients. The recently developed WHO Fungal Priority Pathogens List now identifies critical, high, and medium-priority fungal pathogens ranked based on their public health impact and/or risk of emerging antifungal resistance. For *Candida* species, the WHO critical priority group currently includes *C. albicans* and *C. auris*. Also, *C. glabrata*, *C. parapsilosis*, and *C. tropicalis* are classified as high-priority pathogens, while *C. krusei* is listed in the medium-priority group (Parums, 2022).

There have been reports of an increasing emergence of non-*albicans Candida* species, possibly due to the development of antifungal resistance (Wang et al., 2022) and improved differentiation methods. In our study, *C. tropicalis* (20%), followed by *C. glabrata* (16.76%), *C. krusei* (5.4%), and *C. parapsilosis* (5.08%) were the most prominent non-*C. albicans* species in the respiratory specimens. Other studies have also shown that *Candida* species, particularly *C. albicans*, are commonly isolated from respiratory specimens of the patients. In a study conducted on 100 sputum samples from pulmonary tuberculosis in Mumbai, India, *C. albicans* was identified in 24 out of 26 isolates (92.31%), while *C. tropicalis* and *C. parapsilosis* were isolated from only one case each (Baradkar et al., 2009). In Michigan, *C. albicans* was the most prevalent (78%) species, followed by *C. glabrata* (11%) (Pendleton et al., 2018). Similarly, in Massachusetts, among patients with cystic fibrosis (CF), *C. albicans* (44.6%), *C. tropicalis* (6%), *C. glabrata* (5%), and *C. parapsilosis* (4%) were the most commonly isolated yeasts in respiratory secretions (Azoulay et al., 2006). Therefore, it seems that composition of the species colonizing the respiratory system is consistent with the species responsible for infection leading to hypothesis that *Candida* colonization may serve as a marker/source of patient deterioration.

In COVID-19 patients, immune dysfunction, lung injury, invasive managements including mechanical ventilation, and treatment with various drugs such as antibiotics and corticosteroids contribute to a high risk of colonization or secondary fungal infections (Erami et al., 2023) including invasive candidiasis (Avkan-Oğuz et al., 2022; Froidefond et al., 2023). In the present study, *C. albicans* (n=99, 29.64%) was the most commonly isolated yeast from the RT of COVID-19 patients followed by *C. tropicalis* (n=54, 16.16%) and *C. glabrata* (n=36, 10.77%). Similarly, Avkan-Oguz et al. reported that *C. albicans* (45.5%), *C. glabrata* (15.9%), and *C. parapsilosis* (13.6%) were the most frequently isolated yeasts from critically ill COVID-19 patients who had fungal infection/colonization (Avkan-Oğuz et al., 2022). Likewise, in COVID-19 patients admitted to ICUs with severe SARS-CoV-2 infection in France, *C. albicans* (74%), *C. tropicalis* (8%), *C. glabrata* (6%), *C. dubliniensis* (3%), and *C. parapsilosis* (3%) were most commonly isolated yeast species in respiratory sample cultures (Froidefond et al., 2023). In a study by Erami et al. conducted in Kashan, Iran, among patients with COVID-19 pneumonia who were on mechanical ventilation in ICUs, *C. albicans* (79.7%) was the most prevalent species, followed by *C. glabrata* (17.4%) and *C. africana* (2.9%) (Erami et al., 2022). In our study, 18 cases of co-colonization of *C. albicans* and *C. glabrata* were observed in patients with COVID-19 who received corticosteroid therapy. While previous reports have highlighted the cases of *C. glabrata* pneumonia with candidemia in patients suffering from COPD (Yazici et al., 2016), our study found that *C. tropicalis* was the most common species among COPD patients.

In our study, the most common yeast species identified from 37 hospitalized cancer patients who received anti-cancer therapy was *C. albicans*, which confirms the previous reports (Yu and Liu, 2022). Other *Candida* species, including *C. glabrata*, *C. tropicalis*, *C. krusei*, *C. kefyr*, *C. dubliniensis*, *C. lusitaniae*, and *C. parapsilosis* were also identified in these patients. *C. albicans*, *C. tropicalis*, and *C. glabrata* were similar across all other groups of patients (i.e., COVID-19, cancer, and ICU patients), except for the COPD group, in which the only species were *C. albicans* and *C. tropicalis*.

*Candida auris*, a global nosocomial pathogen with varying antifungal resistance, poses identification challenges, especially in ICU settings. In our study, *C. auris* colonization was detected in three COVID-19 and ICU patients who were hospitalized in the ICU. These patients displayed immunosuppression due to extended ICU stays, multiple complications including severe acute respiratory syndrome coronavirus infection, and the frequent use of high-dose corticosteroid therapy. The presence of *C. auris* appears to correlate with patients’ vulnerability arising from comorbidities, invasive treatments, and prolonged hospitalization. Among the reviewed studies, we encountered a single case of *C. auris* detected in the RT of an ICU-admitted patient in Italy (Corcione et al., 2022). Therapies involving steroids, immunomodulatory agents, and consistent use of broad-spectrum antibiotics might pose risk factors for *C. auris* acquisition, aligning with known risks for invasive *Candida* species infections (Snyder and Wright, 2019). The occurrence of three cases of *C. auris* colonization COVID-19 ICU-admitted in our patients notably emphasizes that immunosuppressed patients with extended hospital stays and multiple complications may present concurrent conditions.

Two strains of *M. capitatus* were isolated from our patient and classified as colonization. *M. capitatus*, previously known as *Geotrichum capitatum* or *Blastoschizomyces capitatus*, is a rare cause of systemic fungal infection in immunocompromised individuals. However, its true frequency is often underestimated, as it is an emerging opportunistic pathogen (Zhu et al., 2022). The prevalence of *M. capitatus* infections has been documented in European countries like Italy, Spain, and France (Mazzocato et al., 2015), and there have been reports highlighting its potential to infect immunocompetent patients as well (Tanabe and Patel, 2018). *M. capitatus* has been found in household dishwashers due to its thermophilic nature, suggesting a potential source of contamination (Zalar et al., 2011). However, there is limited information available from Asia, as there are only a few case reports (Subramanya Supram et al., 2015).

We observed non-*Candida* colonization including *Saccharomyces cerevisiae*, *Trichosporon* spp, *M. capitatus*, and *Nakaseomyces glabratus* in the COVID-19 patients. *Saccharomyces cerevisiae* colonization was found in 3.52% of the COVID-19 patients and 8.69% of ICU/COVID-19 patients, suggesting that ICU hospitalization may provide favorable conditions for *Saccharomyces cerevisiae* growth. *Trichosporon* spp, *M. capitatus*, and *Nakaseomyces glabratus* colonization were observed in approximately 2.5% of the COVID-19 patients.

Yeast isolation from RT samples, such as sputum, BAL, and TA, is a common occurrence, and distinguishing between colonization and infection can be challenging. In our study, none of the colonized patients were proven to have *Candida* lung infection. The significance of a positive culture for *Candida* in severely immunosuppressed patients remains unclear. Currently, the widely accepted criterion for the definitive diagnosis of *Candida* pneumonia is the histologic demonstration of the fungus in lung tissue, accompanied by inflammation (Baum, 1960; De Pascale and Antonelli, 2014).

This study has limitations: 1) The absence of demographic data, patients’ mortality status, duration of hospitalization, cause of death, and short-term follow-up of the patients, primarily due to the critical conditions and high workload during the COVID-19 epidemics. 2) Some yeast colonies had delayed molecular identification, resulting in missing several isolates in subcultures over the course of several months. 3) Antifungal susceptibility testing was not performed for the isolates. In conclusion, *Candida* colonization in the respiratory tracts is a complex issue with varying implications depending on the context. While some studies suggest that it may be associated with worse outcomes in immunocompromised patients and an increased risk of pneumonia, other studies propose that it may be a normal part of the respiratory flora and contribute to community-acquired pneumonia (Pendleton et al., 2017). *Candida* colonization in the RT might act as an independent risk factor promoting ventilator-associated pneumonia and even altering the antibiotic resistance patterns of pathogenic bacteria through polymicrobial biofilm formation (Liu et al., 2021). Airway *Candida* colonization is associated with pulmonary inflammation and subsequent cellular immune dysfunction (De Pascale and Antonelli, 2014). Further research is needed to fully understand the significance of *Candida* colonization in the RT. Although *Candida* pneumonia is rare in both immunocompetent and immunocompromised patients, it is important not to underestimate the clinical implications of *Candida* isolation from the RT, especially in immunodeficient patients.

## Data availability statement

The data presented in the study are deposited in the NCBI (GenBank) repository, accession numbers OR600362, OR600363, OR600282, and OR600243. The original contributions presented in the study are included in the article/supplementary materials. Further inquiries can be directed to the corresponding author.

## Ethics statement

The studies involving humans were approved by The ethics committee of the Isfahan University of Medical Science IR.MUI.MED.REC.1400.448. The studies were conducted in accordance with the local legislation and institutional requirements. Written informed consent for participation in this study was provided by the participants’ legal guardians/next of kin.

## Author contributions

FR: Writing – review & editing, Writing – original draft, Visualization, Software, Methodology, Investigation, Data curation. SaS: Writing – review & editing, Writing – original draft, Visualization, Software, Methodology, Investigation, Data curation. SoS: Writing – review & editing, Resources. EN: Writing – review & editing, Resources. MH: Writing – review & editing, Investigation. SG: Writing – review & editing, Investigation. SA: Writing – review & editing, Visualization, Methodology, Investigation. HF: Writing – review & editing, Resources. HM: Writing – review & editing, Visualization, Supervision, Resources, Project administration, Methodology, Funding acquisition, Data curation, Conceptualization.
